# Effects of cultural adaptation resilience promotion program for mothers-in-law in multicultural families

**DOI:** 10.1371/journal.pone.0274224

**Published:** 2022-09-09

**Authors:** Sang-Hwa Lee, Dong-Hee Kim, Kyoungrim Kang

**Affiliations:** 1 Department of Nursing, Gimhae College, Gimhae-si, Gyeongsangnam-do, South Korea; 2 College of Nursing, Research Institute of Nursing Science, Pusan National University, Yangsan-si, Gyeongsangnam-do, South Korea; Zagazig University Faculty of Human Medicine, EGYPT

## Abstract

Mothers-in-law in multicultural families tend to experience psychological burden. This study aimed to verify the effects of the cultural adaptation promotion resilience program (CAPRP) on resilience, acculturation stress, depression, and quality of life among mothers-in-law in multi-cultural families. Forty-two participants from multicultural family support centres in the metropolitan city A were assigned to either the intervention group or the control group. The CAPRP was performed for 60 minutes, twice a week for four weeks. The intervention group showed a significant decrease in the acculturation stress (*p* = .002), and depression (*p* = .006), while resilience (*p* < .001) and quality of life (*p* < .001) significantly increased compared to the control group. The intervention group reported significant improvements in resilience, acculturation stress, depression, and quality of life in comparison with the control group. The results indicated that the CAPRP, developed based on positive cognitive appraisal, was an efficient nursing intervention for mothers-in-law in multicultural families.

## Introduction

After initiating a couple of decades ago, the percentage of transnational marriages reached the highest percentage at 30.7% in 2005. Although there has been a decrease, it is still at about 10% of the total number of marriages in South Korea, where the current intervention study was conducted [1]. Recently, international marriage had increased, in particular, Korean males who have difficulties in finding their spouses domestically tend to marry foreign females mostly from Southeast Asia, including Vietnam, Cambodia, and Uzbekistan as developing countries [2]. Korea traditionally had homogeneous culture and race [3]. Compared to other countries, such as USA, Canada, and Australia, ethnic diversity in South Korea has not existed long enough to accommodate other cultures easily [4]. Thus, there might be difficulties in accepting other culture among Korean multicultural families, whose family members have different nationalities. A study of Korean mothers-in-law showed that only 11% of them accepted their foreign daughters-in-law’s culture [5]. Taking into account trends of international marriage in Korea [2], multicultural families in the current study refers to as families with Korean husbands and foreign spouses, and mothers-in-law in multicultural families meant Korean mothers on the husbands’ side.

Different from USA, Australia, and European countries, the married couple, a Korean husband and a foreign wife, are more likely to live in together with the Korean male’s parents. Multicultural families with Korean males and foreign females are five times more likely to live with mothers-in-law at 75% than domestic families at 14.1% [6, 7]. Similarly, in the South Asian cultural context, if a mother becomes a widow, she is likely to cohabit with their married son [8]. As Korean culture is different from Southeast Asian culture [9], mothers-in-law of foreign female partners in multicultural families tend to take the role of introducing Korean culture and courtesies including table manners [7]. Specifically, although both Korea and Vietnam, one of the most common countries foreign daughters-in-law are from, have a Confucian background, there are differences in language, cuisine, and culture, including uncertainty avoidance and long-term orientation [10]. For example, Koreans seem to be rigid and intolerant of unwonted or unexpected behaviours or opinions. On the other hand, Vietnamese tend to be more laid-back and flexible towards rules [11]. As seen in the previous literature [12], the elderly mothers-in-law experienced stress due to a burden of supporting their son’s family and the differences in culture from Vietnamese daughters-in-law. Meanwhile, mothers-in-law might have psychological stress due to communication difficulties at more than 30% and cultural differences at slightly under 15% [13]. Moreover, they might be concerned that their foreign daughters-in-law could be running away from their sons and the couple’s grandchildren be left behind, and afraid of blame from their son or others for conflicts with their daughters-in-law [14]. Thus, mothers-in-law of multicultural families may be perceived as three times more stressful than others (F = 18.84, *p* < .001) [7].

Exposure to negative stress and failure of coping strategies can lead to depression, loss of meaning of life, demotivation, and impairment of quality of life (QoL) [15]. Compared to 20% of older adults who generally experience depression [16], mothers-in-law reported depression up to at 84%, including 75% with a high level of depression [5, 6]. Accordingly, they perceive their QoL impaired [14].

According to Lazarus [15], stressed out individuals tend to perform cognitive appraisal consciously or unconsciously in order to minimise physical and/or mental harm by stress. If an individual perceives stress as a threat or realises their absence of stress coping strategies, the level of stress can be more negatively appraised than the actual stress level. A negative appraisal can lead to physiological changes, such as an increase of blood pressure and heart rate acceleration, and can interfere with adaptation to stress. Repeated negative self-assessment can even cause physical illness, anxiety, depression, impaired QoL, and social dysfunction.

On the other hand, a resilient response to stress can lead to flexible coping strategies in various situations [17, 18]. Resilient cognitive appraisal can improve understanding of actions and emotions under stressful situations, acceptability of others, acceptance of diversity, amicable relationships with others, belief and acceptance of life, and optimism for the future [19–21]. Furthermore, it can lead to decreases of cholesterol, anxiety, stress, depression, and increases of belief and acceptance of life and satisfaction [22–25].

Resilience programs help in building psychological flexibility and relaxation [22] and in enhancing the individual’s inner strength so as to adapt to stressors [26]. In most of the previous literature, resilience programs aimed at promoting resilience to negative stress for children [27], college students [20, 24], employees [22], and patients [25, 26]. A resilience program for oncology–haematology nurses in the previous study found to be ineffective for improving their stress management and QoL in the short-term and long-term points of view [28]. However, a resilience program for farmers showed significant effects on their QoL [29]. Although resilience is manifested throughout the individual’s whole life, including older adult life, and is capable of being learned [30], there is scant literature about subjects in old age, particularly in South Korea. In addition, there is a need for developing interventions for cultural adaptation stress, depression, and QoL in Korean mothers-in-law of multicultural families, which have been rarely developed and applied. While foreign daughters-in-law could be well-supported by programs or activities from multicultural family centres, improving resilience of mothers-in-law, who have been accustomed to rigid homogenous culture, would be helpful for accepting the foreign culture and different lifestyle of their daughters-in-law in the globalised era. Therefore, considering the need from the previous literature, this study aimed to develop and implement the cultural adaptation promotion resilience program (CAPRP) for mothers-in-law in multicultural families, and to verify its effects on resilience, acculturation stress, depression and QoL among mothers-in-law in multicultural families, comparing with the control group.

## Methods

### Study design

**Fig 1:**
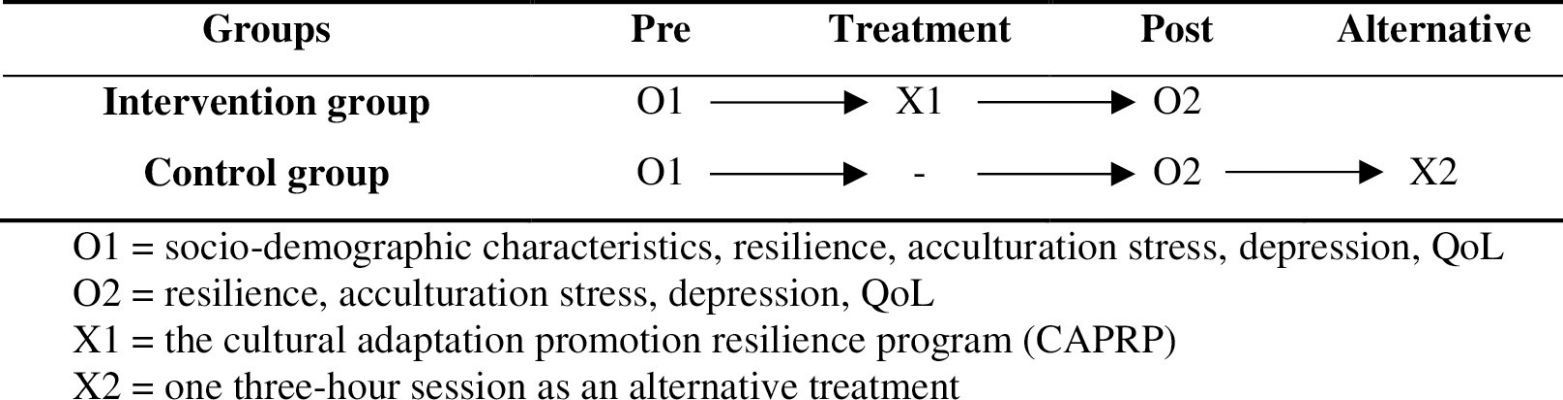
Study design.

A non-equivalent control group pre-posttest design was adopted for evaluating effects of the cultural adaptation promotion resilience program (CAPRP) for mothers-in-law in multicultural families. Both groups completed pre- and post-test questionnaires, but the intervention group participated in the CAPRP. After completion of the study, the control group was provided an alternative program, only one three-hour session as needed. The study design is presented in Fig 1.

### Study subjects

Participants were recruited from multicultural family support centres in the metropolitan city A. A list of nine multicultural centres in the metropolitan city A was found and two centres accepted to participate in the study after the first author either phoned or visited the centres to explain the study’s process. The intervention group was randomly chosen from either C or D centres by picking out one piece of paper from an opaque box. Twenty-four participants from the C centre who consented and were enrolled to the study considered to be in the intervention group, while 25 from the D centre to be in the control group. Inclusion criteria were participants who were 1) Korean mothers-in-law, living with her foreign daughter-in-law more than six months; 2) providing consent to participate in this study; 3) without mental or cognitive impairment; 4) able to understand and answer questions in Korean; and 5) without any hearing impairment.

The sample size of the study was calculated in consultation with a health statistician based on the previous studies [31–33]. The G*Power 3.1.2 program was used for calculation at the two independent group t-test at a power of 0.80, a two-tailed significant level of .05, and the effect size of 0.80 based on the previous research in resilience programs for families of patients with chronic schizophrenia [33], for family caregivers of the elderly with dementia [31], and for trauma-exposed refugees [34]. A sample size of 21 participants in each group was required. Taking into account a dropout rate of 10%, a total of 50 eligible participants were invited in the intervention group and the control group. Among 25 invitees in each group, 21 participants in the intervention group enrolled for the program after three dropped out due to no show at the first day of the program. Of the 25 invitees, 22 participants in the control group agreed to take part in this study. At the follow-up, 21 participants from each group completed the entire study after one in the control group dropped out.

### Instruments

The questionnaires of the study consisted of socio-demographic characteristics [5], resilience [35], acculturation stress [36], depression [37], and QoL [38]. Appropriate permissions from developers of each instrument were received.

#### Socio-demographic characteristics

The socio-demographic characteristics of participants in this study included age, marital status, education level, and religion. Their perceived economic level, health state, and satisfaction of family support was answered on a five-point Likert scale. Information of whether they have any disease was also asked as well as disability due to disease on a three-point Likert scale. Questions about their daughters-in-law included the country which their daughters-in-law were from, duration of living with them, perceived daughters-in-law’s Korean level on a five-point Likert scale, satisfaction with the relationships with daughters-in-law on a five-point Likert scale, and ethnocentrism, asking if their daughters-in-law should fully adapt Korean culture regardless of their original culture, on a three-point Likert scale.

#### Resilience

The Resilience Scale-14 (RS-14), developed by Wagnild & Young [35], was used for assessing resilience of mothers-in-law in multicultural families. This instrument consisted of 14 items, which can be divided into two dimensions—personal competence (10 items) and acceptance of self and life (4 items). Each item is scored on a seven-point Likert scale, ranging from 1 (strongly disagree) to 7 (strongly agree). A range of the total score is between 14 and 98, higher scores indicating higher resilience. Subcategories can be referred to as very low (14–56), low (57–64), moderately low (65–73), moderately high (74–81), high (82–90), very high (91–98). The Cronbach’s α was .93 when developed, and .91 for the current study.

#### Acculturation stress

Acculturation stress of mothers-in-law in multicultural families was measured with a scale developed by Chung& Park [36]. This instrument includes 11 items, which can be divided into three domains—‘perception of prejudice and discrimination’, ‘cultural conflicts’, ‘discomfort with daily life’. Each item is scored on a five-point Likert scale, ranging from ‘strongly disagree’ (1 point) to ‘strongly agree’ (5 points). A range of the total score is between 5 and 55, higher scores indicating higher acculturation stress. The Cronbach’s α was .86 when developed, and .94 for the current study.

#### Depression

Depression of mothers-in-law in multicultural families was measured by the 30-item Geriatric Depression Scale Short Form-Korean Version (GDSSF-K), which Kee [37] standardised and modified for Korean older adults. This instrument consists of 10 positive items and five negative items, which are answered by yes, scored 0, or no, scored 1. A range of the total score is between 0 and 15, higher scores indicating higher depression. The Cronbach’s α was .88 when developed, and .79 for the current study.

#### Quality of life

The Korean Version of WHO Quality of Life Scale Abbreviated Version (K-WHOQOL-BREF) by Min et al. [38] was used to measure QoL of mothers-in-law in multicultural families. This instrument contains 24 items, which can be grouped into physical health (7items), psychological (6 items), social relationship (3 items), and environmental (8 items) domains, as well as two additional items about general QoL and general health perception. Each item is answered by a five-point Likert scale from 1 (strongly disagree) to 5 (strongly agree). The range of the total score is between 26 and 130, higher scores indicating higher QoL. Min et al. [38] reported Cronbach’s α as .89, and the value of Cronbach’s α was .94 for the current study.

### The cultural adaptation promotion resilience program (CAPRP)

#### Preparation for conducting the program

The CAPRP program was developed by the first author for mothers-in-law in multicultural families in this study and provided by the first author and an assistant researcher after appropriate training. For preparing for implementation of the program, the first author had completed several programs of art counselling psychology, positive psychology, resilience coaching, narratives, motivation counselling, and built experiences in developing and conducting a strength-focused resilience promotion program for older women. In addition, the first author tried to expand her expertise through attending various conferences and workshops related to psychology. Two assistant researchers were prepared for data collection by the first author for consistency.

#### Development process and implementation of the program

Goals, components, contents, activities, and the number of sessions of the program were formulated after relevant literature review [30, 31, 39, 40]. Professionals majored in geriatric nursing, psychiatric nursing, and psychiatry as well as doctors of positive psychology and resilience in older adults amended strategies, then approved validity.

Previous literature revealed that major stressors of mothers-in-law in multicultural families were differences in culture and lifestyle from their foreign daughters-in-law, and that they have somatic symptoms, severe depression, impaired QoL, enhanced by passive and negative coping strategies. The final goals of the program thus are ‘improvement in cultural adaptation resilience’, ‘a decrease of cultural adaptation stress’, ‘a decrease of depression’, and ‘an increase of QoL’.

Based on the seven components of resilience, a goal for each session was ‘self-opening’, ‘causal analysis’, ‘impulse control’, ‘emotion regulation’, ‘empathy’, ‘sense of optimism’, and ‘utilisation of strengths’. The contents and other details of the program are presented on Table 1.

**Table 1:** Cultural adaptation promotion resilience program.

Sessions	Subjects	Contents	Strategies	Activities
1	Self-opening	Open mind	Opening the mind by positive image about oneself	Making name tags, greeting
		Building intimacy	Building a comradeship by closeness action	Give nickname, handshake / hugging
		Building supportive relationships through shared experience	Expressing experience after receiving foreign daughters-in-law	Presentation of own experience, support
2	Causal analysis	To recognise cultural differences	Understanding diversity of views	Lecture, drawing a person that everybody knows
		Meaning of difference	Looking for cause by using cases	Lecture and case utilising of picture materials like ‘two people with same clothes & number matching’
		Understanding cultural differences between countries	Checking the culture of daughter-in-law	Lecture
3	Impulse control	Awareness of one’s uncontrollable impulses	Expressing experience of impulsive behaviours	Watching an impulsive behaviour video, watching impulsive behaviour-health related video
		Understanding of impulse	Understanding connection of impulsive behaviour and background belief	ABC theory lecture
		Training of impulse control	Learning conflict-controlled method	Meditation, drawing mandala, tearing a piece of paper
4	Emotion regulation	Awareness of one’s uncontrollable emotions	Recognition difficult situations of own emotion control	Watching a video of emotion explosion
		Experiencing or feeling the uncontrolled emotion of daughters-in-law side	Taking the stance of daughter-in-law	Role play
		Training of emotion control	Relaxing, Singing	Meditation, singing
5	Empathy	Recognising own ineffective communication	Expressing experiences	Presentation
		Understanding the sympathy	Learning sympathetic posture and attitude and efficient communication	Lecture, dialogue picture
		Practicing sympathy	Practicing efficient communication	Role play, writing postcard
6	Sense of optimism	Confirming negative attitude and belief about the situation of one’s stress	Expressing experiences	Presentation, writing on the blackboard
		Understanding the positive and negative expression	Understanding an influence of expression	Watching a video of the power of words, discussion
		Learning optimistic attitude and belief (positiveness training)	Case study, Correcting expression	Watching a video of person who overcame to crisis
7	Utilisation of strengths	Exploring the social resource	Introducing resources of multiple cultures	Lecture
		Exploring personal resources	Finding personal emotion	Presentation, discussion, activity paper
		Using the emotion resource	Planning utilisation of emotion	Presentation

The CAPRP in this study was implemented twice per week for four weeks, 60 minutes per session for a total of seven sessions. The program was provided to 21 participants in the intervention group by the first author of the present study with the help of a pre-trained assistant. An education room in the multicultural centres was selected, considering the participants might be vulnerable, so that the participants was able to feel comfortable and free to share their thoughts. The venue was equipped with internet and a beam projector, used for presenting various examples of multiculturalism.

### Data collection process

Data were collected from January 04, 2016 to March 04, 2016 by two trained research assistants to avoid researcher bias. On the first day of the program, three of the enrolled participants in the intervention group were no-shows, and 21 participants completed the baseline questionnaires and took part in the program. In the control group, 22 participants attended to answer the baseline questionnaires. One participant in the control group dropped out at the follow-up. A total of 42 participants were included in and completed the study. A week after completion of the program, both participants in the intervention group and the control group were asked to answer follow-up questionnaires.

### Ethical considerations

An ethics approval from the Institutional Review Board of the related university was obtained (2015_88_HR). Two research assistants explained the purposes of this study, its procedure, voluntary participation, confidentiality and privacy, then the written informed consent was obtained from all participants. Incentives for participation were provided with the equivalent of transport fares.

### Data analysis

The collected data were analysed using the SPSS/WIN 23.0 program. The homogeneity of the two groups was analysed with descriptive statistics, chi-square test, Fisher’s exact test, and t-test. The effects of the program were examined by t-test after a normality test of the dependent variable and the effect size of the CAPRP was calculated and presented with Cohen’s d (95% confidence intervals) based on resilience, acculturation stress, depression, and QoL in both pre-and post- scores. Statistical significance was set at the level of p < .05 for a two-tailed test.

## Results

### Participant characteristics and homogeneity

Table 2 presents the homogeneity of general characteristics for two groups, which were not significantly different. Age range of the participants in this study was from 62 to 79 years old, with the mean age of 68.48±4.09 in the intervention group and 70.10±5.06 in the control group. Among the participants, 47.6% of the intervention group and 61.9% of the control group were satisfied with the relationship with their daughter-in-law. A majority of participants in both groups—90.5% of the intervention group and 80.9% of the control group—responded that their daughters-in-law should follow the Korean culture.

**Table 2:** Homogeneity test of general characteristics for two group (N = 42).

Characteristics	Categories	Exp.(n = 21)	Cont.(n = 21)	x^2^ or t	*p*
		n (%) or M±SD	n (%) or M±SD		
**Age(yr)**		68.48±4.09	70.10±5.06	1.16	.288
	≤65	7(33.3)	4(19.0)	1.14	.567
	66∼70	7(33.4)	9(42.9)		
	≥71	7(33.3)	8(38.1)		
**Marital state**	Married	11(52.4)	9(42.9)	0.38*	.379
	Others (single, divorced)	10(47.6)	12(57.1)		
**Education level**	None	6(28.6)	3(14.3)	-0.21	.834
	Elementary	9(42.8)	15(71.4)		
	≥Middle school	6(28.6)	3(14.3)		
**Religion**	Have not	6(28.6)	9(42.9)	0.93*	.520
	Have	15(71.4)	12(57.1)		
**Economic level**	Low	3(14.3)	7(33.3)	2.10*	.139
	≥Moderate	18(85.7)	14(66.7)		
**Health state**	Poor	8(38.1)	9(42.8)	0.44	.804
	Moderate	8(38.1)	6(28.6)		
	Good	5(23.8)	6(28.6)		
**Disease**	Have not	7(33.3)	7(33.3)	0.00*	1.000
	Have	14(66.7)	14(66.7)		
**Disability due to disease**	No	9(42.9)	10(47.6)	0.10*	.500
	Yes	12(57.1)	11(52.4)		
**Satisfaction of family support**	Moderate and low	11(52.4)	11(52.4)	0.00*	1.000
	High	10(47.6)	10(47.6)		
**Daughter-in-law’s country**	Vietnam	19(90.5)	13(69.1)	-2.15	.032
	Others	2(9.5)	8(30.9)		
**Duration that daughter-in-law live in Korea (Yr)**	<3	11(52.4)	10(47.6)	0.10*	1.000
	≧3	10(47.6)	11(52.4)		
**Daughter-in-law’s Korean-level**	Low	8(38.1)	6(28.6)	1.05	.593
	Moderate	5(23.8)	8(38.1)		
	High	8(38.1)	7(33.3)		
**Relationship with daughter-in-law**	Moderate & dissatisfaction	11(52.4)	8(38.1)	0.87*	.268
	Satisfaction	10(47.6)	13(61.9)		
**Ethnocentrism**	Disagree	2(9.5)	4(19.0)	-1.89	.058
	Somehow	9(42.9)	13(61.9)		
	Agree	10(47.6)	4(19.0)		

Looking at Table 3, there were no significant differences in scores of baseline questionnaires about resilience, acculturation stress, depression and QoL between the two groups.

**Table 3:** Homogeneity test of dependent variables for two group (N = 42).

Variables	Inv.(n = 21)	Cont.(n = 21)	*t*	*p*
	M±SD	M±SD		
**Resilience**	62.05±12.61	64.57±14.34	-0.61	.548
**Acculturation stress**	32.29±6.41	28.76±11.70	1.21	.235
**Depression**	5.33±3.22	6.00±3.98	-0.60	.175
**Quality of life**	74.62±12.06	71.33±14.90	0.79	.553

Inv. = Intervention group; Cont. = Control group.

### Effects of the CAPRP

The intervention group showed a significant decrease in acculturation stress (t = -3.63, *p* = .002), and depression (t = -3.05, *p* = .006), while resilience (t = 4.04, *p* < .001) and QoL (t = 4.95, *p* < .001) significantly increased compared to the control group. Table 4 presents differences in resilience, stress, and QoL of the intervention group and the control group.

**Table 4:** Differences in variables for intervention and control groups (N = 42).

Variables	Categories	Inv.(n = 21)	Cont.(n = 21)	*t*	*p*	Cohen’s *d* (95% CI)
		M±SD	M±SD			
**Resilience**	Pretest	62.05±12.61	64.57±14.34	-2.52	.548	1.25 (8.78 26.37)
	Posttest	83.29±16.37	68.24±15.87	15.05	.004	
	Difference	21.24±19.57	3.67±3.84	4.04	< .001	
**Acculturation stress**	Pretest	32.29±6.41	28.76±11.70	3.52	.235	-1.12 (-11.64–3.31)
	Posttest	25.10±9.41	29.05±11.27	-3.95	.224	
	Difference	-7.19±9.28	0.29±1.71	-3.63	.002	
**Depression**	Pretest	5.33±3.22	6.00±3.98	-0.67	.553	-0.94 (-3.88–0.78)
	Posttest	3.00±2.51	6.00±3.76	-3.00	.004	
	Difference	-2.33±3.41	0.00±0.84	-3.05	.006	
**Quality of life**	Pretest	74.62±12.06	71.33±14.90	3.29	.437	1.53 (8.82 20.99)
	Posttest	95.52±16.26	77.33±19.62	18.19	.002	
	Difference	20.90±11.59	6.00±7.50	4.95	< .001	

Inv. = Intervention group; Cont. = Control group.

## Discussion

The current study assessed the effects of the seven-session CAPRP on mothers-in-law in multicultural families. The main findings of the current study showed that the CAPRP was effective on improving their resilience and QoL, whereas acculturation stress and depression was diminished. It was difficult to compare the results of this study to the previous research because of the scarce research regarding resilience programs for older females, including mothers-in-law. Conversion scores were used in the discussion, due to differences of instruments in the previous literature.

The participants in the current study (mean age: 70 years old) scored resilience (converted to seven-point scale) as 4.4. This was similar to the study conducted by Kim & Na [41]. The subjects in this study were community dwellers with schizophrenia who have judgement and perception of reality, and reported with the mean score 4.3. On the other hand, patients with neurofibromatosis (mean age: 57 years old) [25], patients with breast cancer (mean age: 60 years old) [26], and Indonesian elders who experienced tsunami (mean age: 66 years old) [19], were more likely to score higher at 4.9, 5.2, and 6.1, respectively, than participants in the current study. The subjects in the studies by Vranceanu et al. [25] and Loprinzi et al. [26] were capable of utilising information about their illness, whereas the subjects in the study by Kim & Na [41] showed a difficulty in perception under a stressful situation. Seemingly, the participants in this study had lower resilience than those in previous studies because they lacked perception of and information about multiculturalism in a single culture society. In addition, not only had they difficulties in daily communication, but also insufficient support and coping resources.

The 5-point converted score of acculturation stress was 2.9 among mothers-in-law in multicultural families in this study. This score was similar to the score 2.8 of Chinese international students in South Korea [42], while it was slightly lower than the score 3.1 of female marriage migrants [43]. The experiences of the participants in the current study were close to those in the previous studies. The Chinese international students expressed such difficulties as ‘feeling alone’, ‘being sick’, and ‘not knowing what to do’ [42]. The marriage migrants showed emotional restraint due to a different cultural point of view [43]. It can be assumed that they might be more stressed out than the international students or the mothers-in-law, requiring multiple roles as a wife, a daughter-in-law, and a mother, as well as acculturation.

As a result of the CAPRP, the 5-point converted score of acculturation stress decreased by 2.3 among the mothers-in-law in multicultural families who used to restrain their emotions in a patriarchal society. Participating in the program, they learned how to express their emotions in a positive way throughout the activities such as drawing a person that everybody knows, drawing a mandala, and tearing a piece of paper. It was considered that the time when they appreciated each other’s drawings might help them understand various points of view. Park & Park [42] applied cognitive art therapy, including drawing and making a story board, to the Chinese international students who had difficulties in verbal communication, resulting in a decrease by 2.4 of acculturation stress. Yang & Lee [43] applied collage group art therapy to the migrant women to help them in recognising their emotions and finding their strengths, resulting in a decrease by 2.0 of acculturation stress. The programs in these previous studies were similar to that in the current study, where participants who had difficulties in verbal expression learned to convey their emotions by drawing activities and spontaneously understood others without blame.

The 15-point converted score of depression was 5.3 (mild depression), showing a decrease to 3.0 after the CAPRP among mothers-in-law in multicultural families in this study. This score was higher than older adults living at home, who scored 1.8 [44]. On the contrary, the score in the current study was lower than patients with neurofibromatosis, scoring 8.4 and 7.6 after the program [25], and with older adults from older citizen’s public centres, scoring 9.7 and 9.0 after the related program [45]. Compared to previous studies, the change in depression in the current study was substantial. It might be due to the fact that the CAPRP enhanced self-perception with causal analysis and acceptability of different cultures, provided enough time for sharing their experiences with the others who were placed in a similar situation. Vranceanu et al. [25] included a problem-based approach for understanding the illness in part of their program; however, the program mainly contained emotional coping strategies, such as meditation and imagination. Lee & Park [45] included in their program notions, causes, and 11 coping strategies for stress, depression, and somatisation. The program ran for five weeks for an hour each week, which might be insufficient for getting acquainted with the information.

After the CAPRP, the 5-point converted score of QoL increased from 2.9 to 3.7 among the mothers-in-law in multicultural families. The results of the current study were similar to those of a study comparing a positive psychotherapy program with a cognitive behaviour therapy program among depressed older adults [46]. The participants in this study showed a 3.0 level of QoL at baseline in both groups. The character strengths-based positive psychotherapy improved QoL to 3.6, while the cognitive behaviour therapy only increased QoL to 3.1 [46].

Similar to the previous studies [23, 46], the program in this study applied coping strategies, such as developing optimism, building positive relationships, and recognising strengths, after identifying negative emotions under stressful circumstances. In addition to this feature, the program also attempted to change circumstantial factors by exposing the participants to positive experiences or positive emotions right after negative experiences, prior to dealing with the culture of their daughter-in-law. As a result of these efforts, the CAPRP was effective in improving participants’ resilience and QoL despite the relatively short research period and the participants’ lower level of demographic characteristics than that of previous studies.

There are several limitations to the present study as follows: Firstly, the mothers-in-law in multicultural families, who participated in the current study, were unable to take part in this study for a longer period because they had to spend much time with house chores and rearing their grandchildren. It is suggested to examine the long-term effectiveness of the program in the future studies. Secondly, comparison with previous studies was limited because there were no previous resilience programs for mothers-in-law in multicultural families. Further studies, applying the CAPRP program with larger sample sizes would be recommended to examine effects of the intervention. Thirdly, the control group did not receive any treatment, so it can be recommended to compare the CAPRP program with other treatments or regular programs for the seniors. In addition, the effect of a program may decrease over time [47], thus a regular cognitive behaviour training program would be suggested for enhancing or maintaining their resilience. Fourthly, we only included mothers-in-law in the program and did not include much information about daughters-in-law’s characteristics, such as age, children in the current marriage, and rural/urban residence before immigration to Korea. It is recommended to develop an integrated program for promoting cultural adaptation of both mothers and daughters-in-law in multicultural families together, taking into account daughters-in-law’s individual characteristics. Lastly, the nationalities of the daughters-in-law of the intervention group were Vietnam at 90%, whereas those of them of the control group were at 69% due to random allocation. Considering the results of the present study, a cautious interpretation is needed. Future studies on effects of the program for different mothers-in-law, who have daughters-in-law from various countries, would be recommended.

In spite of these limitations, the current study has a few strengths. To our knowledge, this CAPRP is the first program for cultural adaptation of older females, that is, mothers-in-law. Previous programs for the elderly were mostly focusing on their depressive symptoms [32]. Recently, a leisure program [48] or a mourning program using art therapy was applied [49]. On the other hand, the CAPRP could enhance participants’ psychological strength comprehensively based on the Lazarus, compared to other programs dealing with only limited aspects. Considering that the communication problem was one of the most important issues for them, the program contained sessions for improving communication skills. It also helped to build on their empathy with their daughters-in-law from different culture by watching videos about multicultural families, particularly about foreign female migrants, and writing cards to their daughters-in-law. Based on a thorough literature review of previous domestic and international studies, the components of resilience among mothers-in-law in multicultural families were identified as self-opening, causal analysis, impulse control, emotion regulation, empathy, sense of optimism, and utilising of strengths. This finding can be applied when developing resilience-related education programs for those with similar characteristics. Additionally, the CAPRP was effective for decreasing stress and depression as well as increasing QoL, thus it can be utilised for intervening in situations of stress, depression, and QoL of the older members of the community, given the fact that the numbers of the older population and migrants are expected to continuously increase until 2040 [50]. The program mostly adopted visual aids as educational materials in order to minimise the limitation of literacy, compared to the previous resilience promotion programs for people without cognitive impairment and illiteracy. Further, the CAPRP could be applied to older females in other countries, whose culture might be as rigid as Korea, to help them become accustomed to different heritage, values, or lifestyle. The program may also be applied to where there is a huge generation gap in any society.

## Conclusions

This study aimed at developing and applying the CAPRP to mothers-in-law who have foreign daughters-in-law, and to identify the effects of the program. The intervention group reported significant improvements in the resilience, acculturation stress, depression, and QoL in comparison with the control group. The results indicated that the CAPRP, developed on the basis of positive cognitive appraisal, was an effective nursing intervention for mothers-in-laws in multicultural families. The visual materials used in this program were efficient enough for the older adults to expand the eligibility of the program.
